# The good, the bad and the dubious: VHELIBS, a validation helper for ligands and binding sites

**DOI:** 10.1186/1758-2946-5-36

**Published:** 2013-07-29

**Authors:** Adrià Cereto-Massagué, María José Ojeda, Robbie P Joosten, Cristina Valls, Miquel Mulero, M Josepa Salvado, Anna Arola-Arnal, Lluís Arola, Santiago Garcia-Vallvé, Gerard Pujadas

**Affiliations:** 1 Grup de Recerca en Nutrigenòmica, Departament de Bioquímica i Biotecnologia, Universitat Rovira i Virgili, Campus de Sescelades, C/ Marceŀlí Domingo s/n, Tarragona, Catalonia 43007, Spain; 2 Department of Biochemistry, Netherlands Cancer Institute, Plesmanlaan 121, Amsterdam 1066 CX, The Netherlands; 3 Centre Tecnològic de Nutrició i Salut (CTNS), TECNIO, CEICS, Avinguda Universitat 1, Reus, Catalonia 43204, Spain

**Keywords:** Electron density map, Binding site structure validation, Ligand structure validation, Protein structure validation, PDB, PDB_REDO

## Abstract

**Background:**

Many Protein Data Bank (PDB) users assume that the deposited structural models are of high quality but forget that these models are derived from the interpretation of experimental data. The accuracy of atom coordinates is not homogeneous between models or throughout the same model. To avoid basing a research project on a flawed model, we present a tool for assessing the quality of ligands and binding sites in crystallographic models from the PDB.

**Results:**

The Validation HElper for LIgands and Binding Sites (VHELIBS) is software that aims to ease the validation of binding site and ligand coordinates for non-crystallographers (i.e., users with little or no crystallography knowledge). Using a convenient graphical user interface, it allows one to check how ligand and binding site coordinates fit to the electron density map. VHELIBS can use models from either the PDB or the PDB_REDO databank of re-refined and re-built crystallographic models. The user can specify threshold values for a series of properties related to the fit of coordinates to electron density (Real Space R, Real Space Correlation Coefficient and average occupancy are used by default). VHELIBS will automatically classify residues and ligands as *Good*, *Dubious* or *Bad* based on the specified limits. The user is also able to visually check the quality of the fit of residues and ligands to the electron density map and reclassify them if needed.

**Conclusions:**

VHELIBS allows inexperienced users to examine the binding site and the ligand coordinates in relation to the experimental data. This is an important step to evaluate models for their fitness for drug discovery purposes such as structure-based pharmacophore development and protein-ligand docking experiments.

## Background

The 3D structure of proteins depends on their amino acid sequence [1] but cannot be predicted based solely on that sequence, except for relatively small proteins [2]. As the structure of a molecule cannot be observed directly, a model of the structure must be constructed using experimental data. These data can be obtained through different methods, such as X-ray crystallography, NMR spectroscopy or electron microscopy. However, none of these methods allows for the direct calculation of the structure from the data. In X-ray crystallography, the most widely applied method, the crystallographic diffraction data are used to construct a three-dimensional grid that represents the probability for electrons to be present in specific positions in space, the so-called electron density (ED) map. The ED shows the average over many (typically between 10^13^ and 10^15^) molecules arranged in a periodic fashion in crystals and is the average over the time of the X-ray experiment [3]. This ED is then interpreted to construct an atomic model of the structure. The model is just a representation of the crystallographic data and other known information about the structure, such as the sequence, bond lengths and angles. Different models, such as the thousands of models in the Protein Data Bank (PDB) [4], represent the experimental data with varying degrees of reliability, and the quality of experimental data (for example, the resolution limit of the diffracted X-rays) varies significantly.

Due to the interpretation step during modeling, which is inevitably subjective [5,6], it is very important to see if a model fits reasonably to the ED that was used to construct it, to ensure its reliability. For drug discovery and design purposes, the model quality of the protein binding sites and of the ligands bound to them are of particular interest, while the overall model quality or the quality of the model outside the binding site are not directly relevant.

A good way to assess how well a subset of atomic coordinates fits the experimental electron density is the Real Space R-value (RSR) [7], which has been recommended by the X-ray Validation Task Force of the Worldwide PDB [8,9]. The RSR measures a similarity score between the 2mFo-DFc and the DFc maps. The real-space correlation coefficient (RSCC) [6] is another well-established measure of model fit to the experimental data. The use of the ED to validate the model will not catch all possible problems in the model [10], but it can show whether the model fits the data from which it was created.

VHELIBS aims to enable non-crystallographers and users with little or no crystallographic knowledge to easily validate protein structures before using them in drug discovery and development. To that end, VHELIBS features a Graphical User Interface (GUI) with carefully chosen default values that are valid for most situations but allows parameters to be easily tuned for more advanced users. A tool named Twilight [11,12] has recently been published to evaluate ligand density. However, while VHELIBS focuses on assessing both the ligands and binding sites to aid model evaluation for drug discovery purposes, Twilight is ligand-centric and focuses on highlighting poorly modeled ligands. VHELIBS also enables the user to choose between the models from either the PDB [4,13] or the PDB_REDO [14] databanks. Using PDB_REDO as the data source can have substantial benefits over using the PDB. PDB_REDO changes models both by re-refinement, incorporating advances in crystallographic methods since the original structure model (the PDB entry) was constructed, and by limited rebuilding, mainly of residue side chains [15], improving the fit of models to the ED [16].

## Implementation

VHELIBS validates the binding site and ligand against the ED in a semi-automatic way, classifying them based on a score of *Good*, *Bad* or *Dubious*. This score is calculated by taking several parameters into account (RSR, RSCC, and average occupancy by default, but more can be used). After performing the automatic analysis and classification of a target’s binding site and ligand, it then enables the user to graphically review and compare them with their ED in order to make it easier to properly classify any structure labeled ‘dubious’ or to re-classify any other structure based on actual visual inspection and comparison of the ED with the model.

VHELIBS is mainly implemented using Python under Jython [17], with some critical parts implemented in Java. It uses Jmol [18] for the 3D visualization of models and EDs. Electron density maps are retrieved from the EDS [19,20] or from the PDB_REDO databank, which are updated weekly with new data from the PDB. Models are downloaded from either the PDB or PDB_REDO according to the user settings.

### Description of the algorithm

VHELIBS takes as input a user-provided list of either PDB [13] or UniProtKB [21] codes (which are mapped to their corresponding PDB codes). The codes in these lists can be entered directly from the GUI or provided in a text file.

For each of these PDB codes, statistical data are retrieved from the EDS or from the PDB_REDO, depending on the source of the models being analyzed (i.e., EDS data for models downloaded from the PDB and PDB_REDO data for models downloaded from the PDB_REDO). Ligands bound with residues or molecules included in the ‘blacklist’ exclusion list (see below) with a bond length < 2.1 Å are rejected. Those ligands bound to molecules in the ‘non-propagating’ exclusion list (which can be modified by the user and by default contains mainly metal ions) are not rejected. The exclusion lists are composed of the most common solvent molecules and other non-ligand hetero compounds often found in PDB files, as well as some less common solvents and molecules that were found to have very simple binding sites (e.g., a binding site consisting of just 1–2 residues). We also incorporated the buffer molecules from Twilight’s list [11,12]. The exclusion list from BioLip [22] was also considered, but deemed too restrictive.

**Figure 1:**
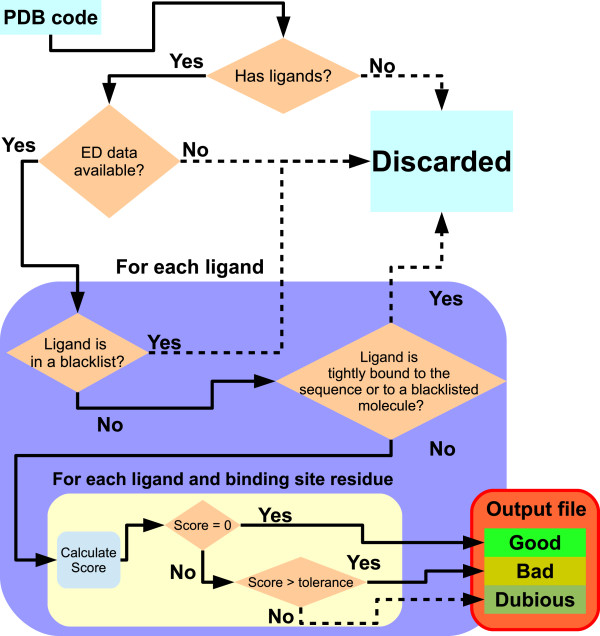
**Automatic ligand and binding site classification.** This diagram shows the process by which the ligands and binding sites of each PDB/PDB_REDO model are classified based on how well the model fits the ED.

Once the ligands are determined, all the residues nearer than a specified distance (4.5 Å by default) are considered to be part of the binding site of that ligand. Then, every ligand and binding site residue is given a score and classified by that score based on the following algorithm (see also Figure 1):

● For each residue and component of each ligand and each binding site, the initial score is defined to be 0.

● For each unmet user-specified condition, the score is increased by 1. The user specified conditions are the value thresholds for several different properties of the model and the data (i.e., RSR, RSCC, occupancy-weighted B factor, R-free, resolution and residue average occupancy; the user may also use a subset of these properties).

● If the score remains 0, the ligand/residue is labeled as *Good.*

● If the score is greater than the user-defined tolerance value, the ligand/residue is labeled as *Bad.*

● If the score is between 0 and the user-defined tolerance value, the ligand/residue is labeled as *Dubious.*

● At the end of all evaluations, the binding site and the ligand (for ligands with more than 1 ‘residue’, i.e., those composed of more than one hetero compound in the PDB file) are labeled according to the worst score of their components (i.e., a binding site with a *Bad* residue will be labeled as *Bad* regardless of how the rest of the residues are labeled, and a binding site can only be labeled as *Good* when all its residues are *Good*).

The results from this classification are saved to a CSV file (the results file), which can be opened by any major spreadsheet software and can then be filtered as desired (for *Good* ligands, for *Good* binding sites or for both). A file with a list of all the rejected PDB structures and ligands and the reason for the rejection is also generated with the results file.

**Figure 2:**
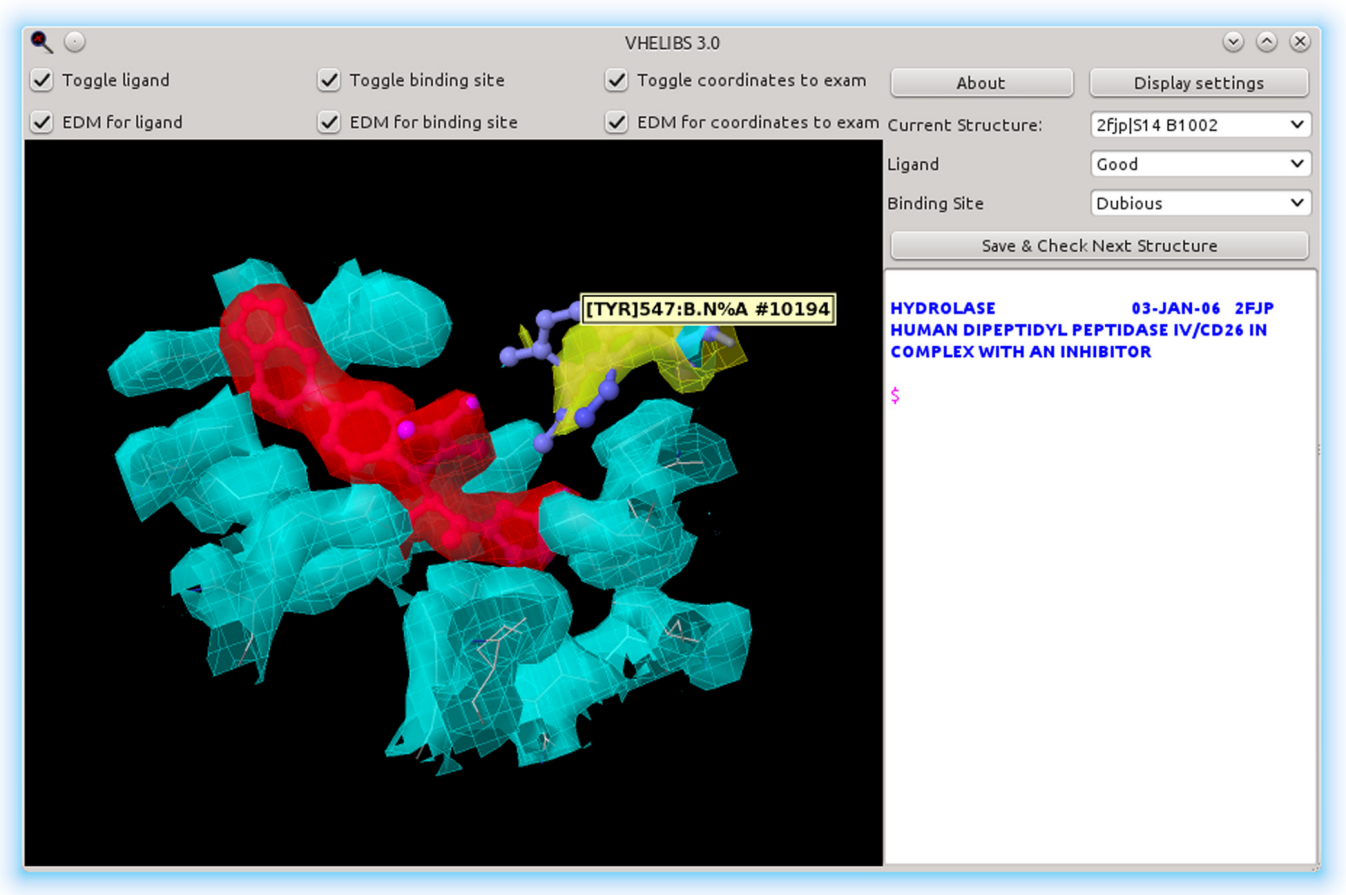
**Example of a Good ligand with a Dubious binding site.** Here, we can see a ligand (S14 B1002 in PDB entry 2FJP [23]) and its binding site, from the analysis of DPP4_HUMAN using the *Default* (PDB) profile. The only dubious residue from the binding site is the one with the yellow ED represented as ball and stick and colored by B-factor.

**Figure 3:**
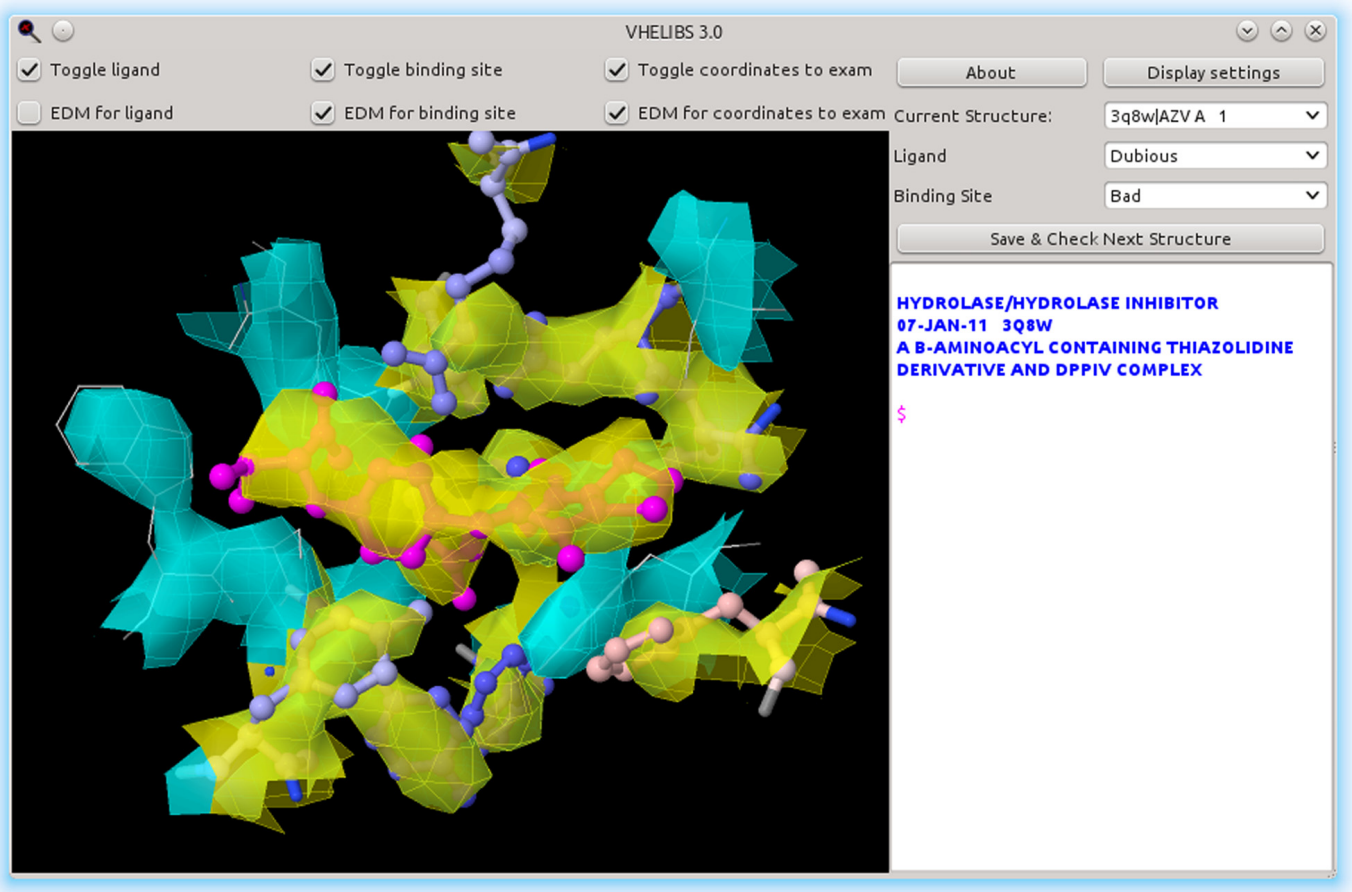
**Example of a dubious ligand with a bad binding site.** Here, we can see a ligand (AZV A 1 in PDB entry 3Q8W [24]) and its binding site from the same analysis as in Figure 2. As can be seen, some residues from this binding site hardly fit their ED (in yellow). The ligand mostly fits its ED, but it still has some discrepancies.

After this automatic classification of ligands and binding sites is complete, the user can visually inspect the results in order to see whether a binding site or ligand labeled as *Dubious* can actually be marked as *Good* (Figures 2 and 3). The default visualization settings provide users of VHELIBS with the appropriate frame to easily reclassify *Dubious* residues and ligands either as *Good* or *Bad*:

● binding site residues are shown by default in white and with a wireframe style in order to show the context where the possible reclassification is evaluated.

● coordinates to examine for veracity are shown in ball and stick style and colored according to their B-factor.

● ligand coordinates are shown in ball and stick style and colored in magenta (but can be colored according to their B-factor if they need to be examined).

● the ED for coordinates to examine is shown in yellow.

● the ED for the complete binding site can be added to the visualization (in cyan) if necessary.

● the ED for the ligand can be shown separately (in red).

Hence, with this visualization frame, the user has all the information he/she needs in order to decide, for instance, whether (a) dubious binding site coordinates could be relevant for protein-ligand docking results (if the dubious coordinates face away from the ligand, it is reasonable to think that their accuracy does not affect protein-ligand docking results); and (b) ligand coordinates that were classified as *Bad* or *Dubious* by the automatic analysis can be changed to *Good* if the experimental pose is the only possibility for its corresponding ED (this can occur with non-flexible rings that have only partial ED for their atoms). In the online documentation (https://github.com/URVnutrigenomica-CTNS/VHELIBS/wiki) [25], there is more information on this and some practical rules for guiding such an evaluation. Of course, the visualization of the binding site, the ligand and coordinates to examine (dubious or bad residues and ligands) and their respective EDs can be customized in several ways through the GUI, e.g., by changing atom colors and styles or the contour level and radius of the EDs.

VHELIBS can be used with different running conditions (i.e., with different *profiles*). The values of the default profiles [i.e., *Default (PDB)* and *Default (PDB_REDO)*] were chosen after careful visualization and comparison of models with their EDs, giving a default minimum RSCC of 0.9, a minimum average occupancy of 1.0, a maximum RSR of 0.4 and a maximum good RSR of 0.24 for PDB and 0.165 for PDB_REDO. The different RSR cut-offs for the PDB and PDB_REDO are the result of RSR being calculated using different software in the EDS (which uses MAPMAN [26]) and in PDB_REDO (which uses EDSTATS [27]). The third provided profile, *Iridium*, is based on the values used in the construction of the Iridium set [28]. This profile is only provided as an example of how easy it is to adapt VHELIBS to use other values found in the literature. Note however that VHELIBS will yield slightly different results from those in the Iridium set, because VHELIBS uses the EDs and statistical data from EDS or PDB_REDO, while the authors of the Iridium set calculate all the data using different software and different EDs.

### Key features of VHELIBS

● Many different parameters can be used to filter good models, and their threshold values can be adjusted by the user. Contextual help informs the user about the meaning of the different parameters.

● VHELIBS comes with three profiles, and the user can create custom profiles and export them for further use or sharing.

● VHELIBS has the ability to work with an unlimited number of PDB or UniProtKB [29] codes (all the PDB codes in each UniProtKB entry are analyzed).

● VHELIBS has the ability to choose between models from PDB_REDO or from the PDB.

● VHELIBS runs in the Java Virtual Machine, which makes it operating-system independent.

● VHELIBS consists of a single jar file, needing no installation. There are no dependencies other than Java.

● The user can load a results file from a previous analysis; one can let a huge analysis run during lunch or overnight and then review the results at any later time.

● A user does not need to be familiar with any other software (although familiarity with Jmol [18] will help the user to make sophisticated custom views).

### PDB_REDO changes to support VHELIBS

The PDB_REDO databank was upgraded to have per-residue RSR and RSCC values and downloadable EDs in the CCP4 [30] format for each entry. These ready-made maps make electron density visualization possible not only in VHELIBS but also in PyMOL [31] (for which a plugin is available via the PDB_REDO website).

To assess how much of the previously observed model improvement in PDB_REDO [16]⁠ is applicable to ligands and their binding pocket, we implemented two new ligand validation routines in the PDB_REDO pipeline: (1) EDSTATS [27] calculates the fit of the ligand with the ED; and (2) YASARA [32] calculates the heat of formation of the ligand (which is used as a measure of geometric quality) and the interactions of the ligand with its binding pocket. The interactions measured in YASARA include the number of atomic clashes (bumps), the number and total energy of hydrogen bonds, and the number and strength of hydrophobic contacts, π-π interactions, and cation-π interactions. The strengths of hydrophobic contacts, π-π interactions, and cation-π interactions are based on knowledge-based potentials [33] in which each individual interaction has a score between 0 and 1.

## Results and discussion

**Figure 4:**
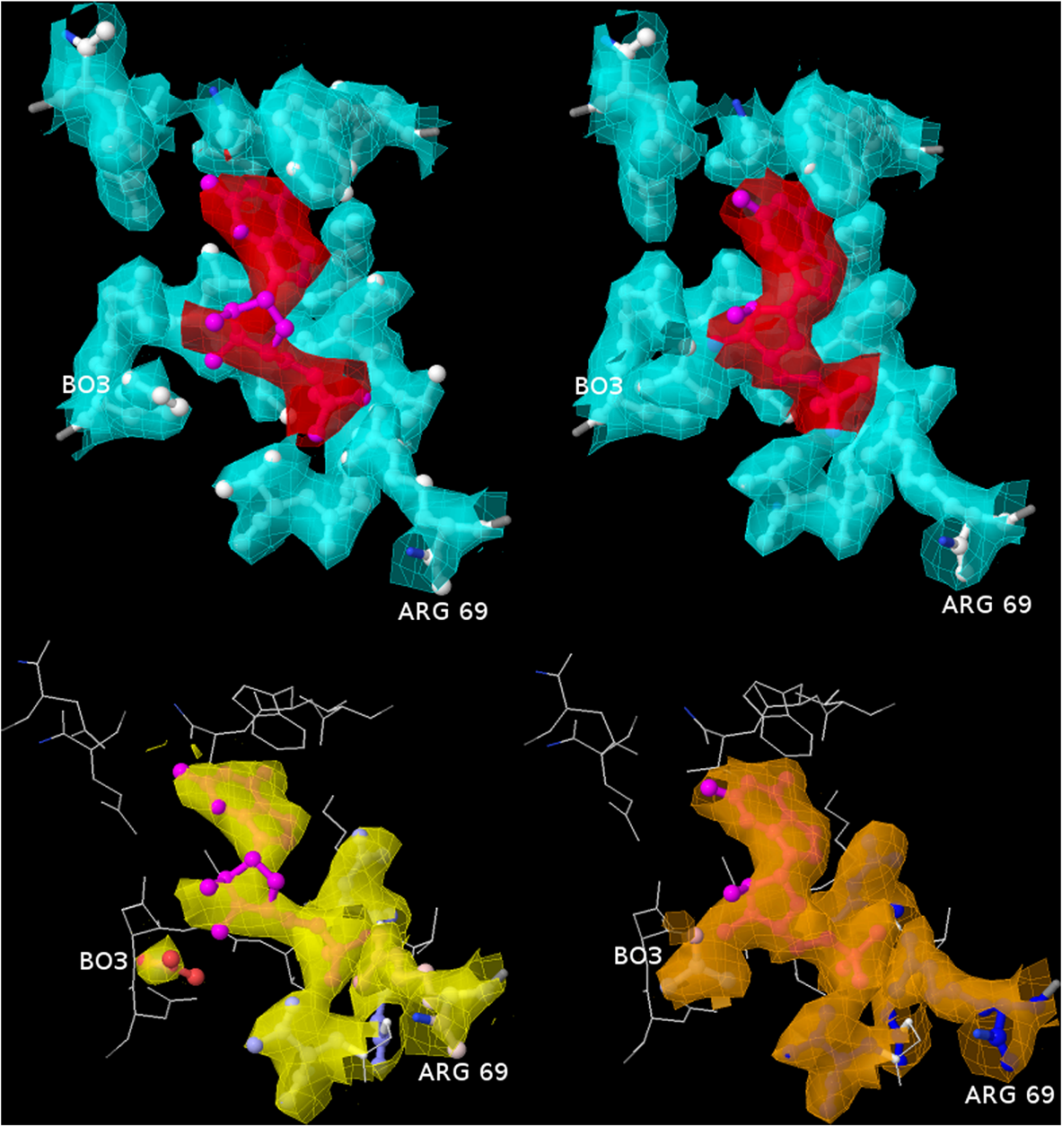
**The guanosine-5′-monophosphate binding site in chain C of PDB entry 1A97 [**[34]**] is an example of a ligand and binding site flagged as dubious and bad in the PDB, respectively (left panel: upper with cyan ED for the binding site and red ED for the ligand; lower with default view: yellow ED for *Dubious *and *Bad *residues), but scored as *Good *in PDB_REDO (right panel: upper with cyan ED for the binding site and red ED for the ligand; lower with previously bad or dubious residues with orange ED).** The RSR and RSCC of the ligand improve from 0.154 to 0.065 and from 0.86 to 0.97, respectively. Two extra hydrogen bonds are introduced, improving the total hydrogen bonding energy from −157 kJ/mol to −199 kJ/mol. The all-atom root mean square deviation of the ligand is 0.6 Å. Of the residues in the binding site, arginine 69 and the boric acid molecule improve most significantly in terms of fit to the ED.

We performed an analysis of the ligand quality scores in the PDB and PDB_REDO for more than 16,500 ligands (compounds described by the PDB as a ‘non-polymer’ and not chemically linked to the protein, with common crystallization additives, such as sulfate and glycerol, excluded) in more than 5,900 structures, and the results are summarized in Table 1. The results show that ligands in PDB_REDO are better in terms of fit to the ED (better RSR and RSCC) and have more favorable geometry (lower heat of formation). Although the interactions with binding sites improve, the changes are very small, except for the reduction in atomic clashes. This is to be expected, as ligand binding sites are typically the most important part of a structure model, and much attention is paid to ensure that the model is correct in that area. Nevertheless, in individual cases the improvement can be great enough to change a *Dubious* ligand in a *Bad* binding site to a *Good* ligand in a *Good* binding site (Figure 4).

**Table 1:** Average validation scores for ligands in PDB and PDB_REDO

**Validation score **^**a**^	**PDB average **^**b**^	**PDB_REDO average **^**b**^
RSR ^c^	0.120	0.104
RSCC ^c^	0.90	0.92
Heat of formation (kJ/mol) ^d^	−1011	−1067
Hydrogen bonding energy (kJ/mol) ^d^	−57.7	−58.8
Hydrophobic contact strength ^d,e^	16.20	16.43
π-π interaction strength ^d,e^	1.26	1.28
Cation-π interaction strength ^d,e^	1.17	1.19
Number of atomic clashes ^d^	9.1	7.9

^a^ A smaller value is better for RSR, heat of formation (strained ligand conformations give higher values), hydrogen bonding energy and number of atomic clashes. A larger value is better for RSCC, hydrophobic contact strength, π-π interaction strength and cation-π interaction strength.

^b^ Average over 16,904 ligands (13,703 for heat of formation) in 5,932 structure models.

^c^ Calculated using EDSTATS [27].

^d^ Calculated using YASARA [32] using the atomic coordinates as is.

^e^ The average reported is the average sum of all interactions for a single ligand.

All ligands and binding sites present in both the EDS and the PDB_REDO databanks were analyzed using the appropriate default profiles [*Default (PDB)* and *Default (PDB_REDO)*]. The results are summarized in Table 2 (for the binding sites) and Table 3 (for the ligands). In the case of the binding sites, the *Good* binding sites in the EDS account for 19%, while in PDB_REDO, they account for 36%, although only 67% of the *Good* binding sites in the EDS are classified as *Good* for PDB_REDO, and some of them are even classified as *Bad*. In the case of the ligands, however, the improvement in classification from the PDB_REDO is far more significant: *Good* ligands increase from 31% from the EDS to 64% from PDB_REDO, with most of the *Good* ligands from the EDS still classified as *Good* from PDB_REDO (95%); *Bad* ligands are dramatically reduced from 43% for EDS to 4% from PDB_REDO, having most of these *Bad* ligands from EDS classified as *Good* from the PDB_REDO. Interestingly, our results suggest that by default, a typical VHELIBS user should choose the *Default (PDB_REDO*) profile instead of the *Default (PDB)* one. From the 16,830 binding sites that are labeled as *Good* by either of the default profiles, 85% of them are identified by the *Default (PDB_REDO)* profile [in contrast with only 46% being identified by the *Default (PDB)* profile]. This is even more remarkable when the ligands are considered: from the 26,028 ligands labeled as *Good* by either of the default profiles, 97% of them are identified by the *Default (PDB_REDO)* profile, and only 48% are identified by the *Default (PDB)*.

**Table 2:** Analysis of all binding sites present in both PDB and PDB_REDO

		**PDB_REDO**			
		**Good**	**Bad**	**Dubious**	
EDS	Good	5,145	1,600	926	7,671
	Bad	5,500	3,727	8,395	17,622
	Dubious	3,659	2,953	7,915	14,527
		14,304	8,280	17,236	39,820

This table shows how binding sites were classified when coming from the EDS or from the PDB_REDO databank.

**Table 3:** Analysis of all ligands present in both PDB and PDB_REDO

		**PDB_REDO**			
		**Good**	**Bad**	**Dubious**	
EDS	Good	11,741	16	662	12,419
	Bad	9,819	1,206	6,098	17,123
	Dubious	3,790	229	6,259	10,278
		25,350	1,451	17,236	39,820

This table shows how ligands were classified when coming from the EDS or from the PDB_REDO databank.

To demonstrate how VHELIBS can be used, we chose as a test case the human Dipeptidyl peptidase 4 (DPP-IV). We first used the corresponding UniProtKB name, DPP4_HUMAN, with the *Default (PDB_REDO*) profile. There are 74 different PDB structures listed in the UniProtKB entry for this protein. The automatic analysis of all of these structures took an average of 2 min 0.43 s on an AMD FX-8150 machine running Ubuntu 12.04.1 LTS amd64 and Java (OpenJDK) 1.6.0_24, with some of the time spent downloading data from the PDB_REDO (with cached PDB_REDO data, and thus without downloading it, the average is 1 min 15.78 s). Out of the original 74 PDB structures, 10 were rejected because there were no PDB_REDO data available for them (1J2E, 1NU6, 1NU8, 1R9M, 1R9N, 1RWQ, 1WCY, 2BUB, 2JID and 2QKY). Rejection occurs most often when a PDB entry lacks experimental X-ray reflection data, which is the case for the ten structures listed. From the remaining 64 structures, 44 had no ligands, leaving 20 structures. These 20 PDB_REDO models showed 450 possible ligand-binding site pairs, of which 9 were rejected because the ligand was covalently bound to a residue, and 366 were rejected because the ligand was either on the exclusion list or covalently bound to a ligand on that list. Most of these rejected ligand-binding sites include molecules such as sulfate/SO_4_, which are marked as hetero compounds by the PDB, covalently bound ligands (e.g., mannose/MAN in 2BGN), or metal ions (e.g., sodium or mercury) that are not usually used for drug discovery purposes. There were 75 valid ligand-binding site pairs. Of these, 55 were labeled as *Good* ligands, 57 as *Good* binding sites and 43 as *Good* ligand and binding site (Table 4). With 55 *Good* ligands and 57 *Good* binding sites (43 of them being *Good* binding sites with *Good* ligands), there should be enough *Good* structures for most uses; it would not be necessary to review the *Dubious* ones. However, if this were not the case, the user could review *Dubious* cases to validate them for the specific purposes. Figure 2 shows one example of a *Good* ligand with a *Dubious* binding site, whereas Figure 3 shows a *Dubious* ligand with a *Bad* binding site. The user can also review the *Good* structures if he or she is looking for false positives, or review the *Bad* ones in the hope of finding good enough structures there (which is very unlikely using the default profiles).

**Table 4:** **Number of complexes classified as *Good*, *Bad *or *Dubious *after applying VHELIBS to 75 ligand/DPP-IV binding site complexes using the *Default (PDB_REDO) *profile**

		**Binding site**			
		**Good**	**Bad**	**Dubious**	
Ligand	Good	43	0	12	55
	Bad	0	0	0	0
	Dubious	14	0	6	20
		57	0	18	75

There are several cases where VHELIBS can prove very helpful:

● VHELIBS can be used to choose structures to use for a protein-ligand docking: with VHELIBS, the user can choose the structures with the best-modeled binding sites.

● VHELIBS can be used to choose structures where both the binding site and the ligand are well modeled, in order to validate the performance of different protein-ligand docking programs. This could make it possible to obtain a new gold standard for protein/ligand complexes that could be used for the validation of docking software and that could be significantly larger and more diverse than those currently being used (i.e., the Astex Diverse Set [35] and the Iridium set [28]).

● VHELIBS can be used to choose structures where both the binding site and the ligand are well modeled to obtain reliable structure-based pharmacophores that select the relevant target bioactivity-modulating intermolecular interactions. This is important in drug-discovery workflows for finding new molecules with similar activity to the co-crystallized ligand.

● VHELIBS can be used to obtain well-modeled ligand coordinates in order to evaluate the performance of 3D conformation-generator software that claims to be able to generate bioactive conformations.

## Conclusions

VHELIBS allows the user to easily check the fit of models to the ED for binding sites and ligands without additional scripting or console commands for each structure. Moreover, our study allows us to conclude that in general, binding site and ligand coordinates derived from PDB_REDO structures are more reliable than those obtained directly from the PDB and therefore highlights the contribution of the PDB_REDO database to the drug-discovery and development community.

## Availability and requirements

**Project name:** VHELIBS (Validations Helper for Ligands and Binding Sites).

**Project home page:**http://urvnutrigenomica-ctns.github.com/VHELIBS/

**Operating System(s):** Platform independent.

**Programming language:** Python, Java.

**Other requirements:** Java 6.0 or newer, internet connection.

**License:** GNU AGPL v3.

**Any restrictions to use by non-academics:** None other than those specified by the license (same as for academics).

## Abbreviations

ED: Electron density; PDB: Protein data bank; GUI: Graphical user interface; RSR: Real space residual; RSCC: Real space correlation coefficient; DPP-IV: Dipeptidyl peptidase 4.

## Competing interests

The authors declare that they have no competing interests.

## Authors’ contributions

ACM, SGV, and GP designed the software and prepared the manuscript. RPJ advised on default parameters, enabled the use of PDB_REDO in VHELIBS and contributed to the manuscript. Testing was performed and feedback for new ideas and GUI design were given by MJO, RPJ, CV, MM, MJS, AAA and LA. The software implementation was performed by ACM with the help of MJO. All authors read and approved the final manuscript.
